# Supplementary figures and images for: Influence of coral and algal exudates on microbially mediated reef metabolism
Source: PeerJ. 2013 Jul 16;1:e108. doi: 10.7717/peerj.108 (PMC3719129; doi:10.7717/peerj.108)

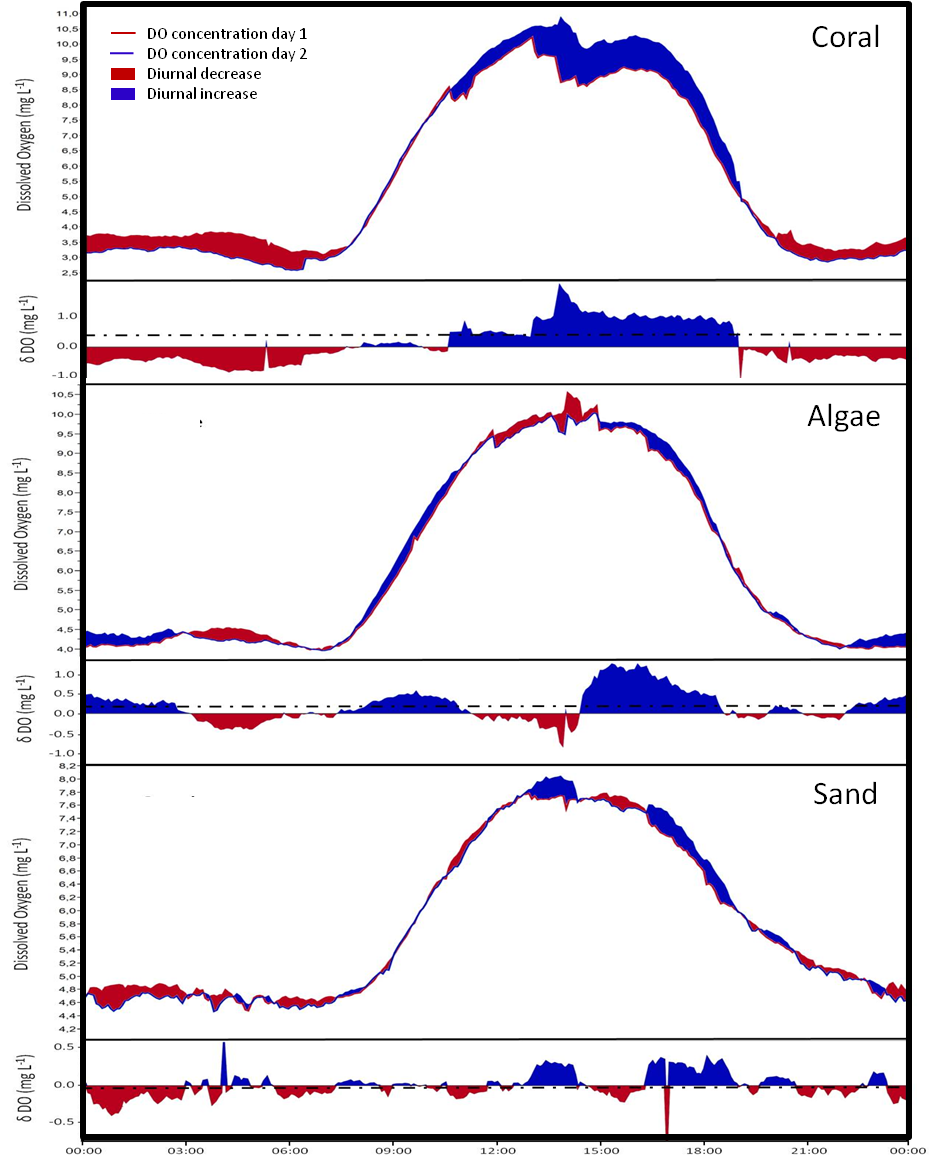

Supplement: Figure S1 — Examples of DO fluxes assessed by autonomously working data loggers in cBITs over a 48 h period. Red line shows values from 0–24 h, blue line from 24–48 h. Decreases over 24 h at a given time point are highlighted by a red area, increases by blue. Lower panels show change at each the respective daytime over 24 h. Mean change is indicated by the dotted line. [file peerj-01-108-s001.png]

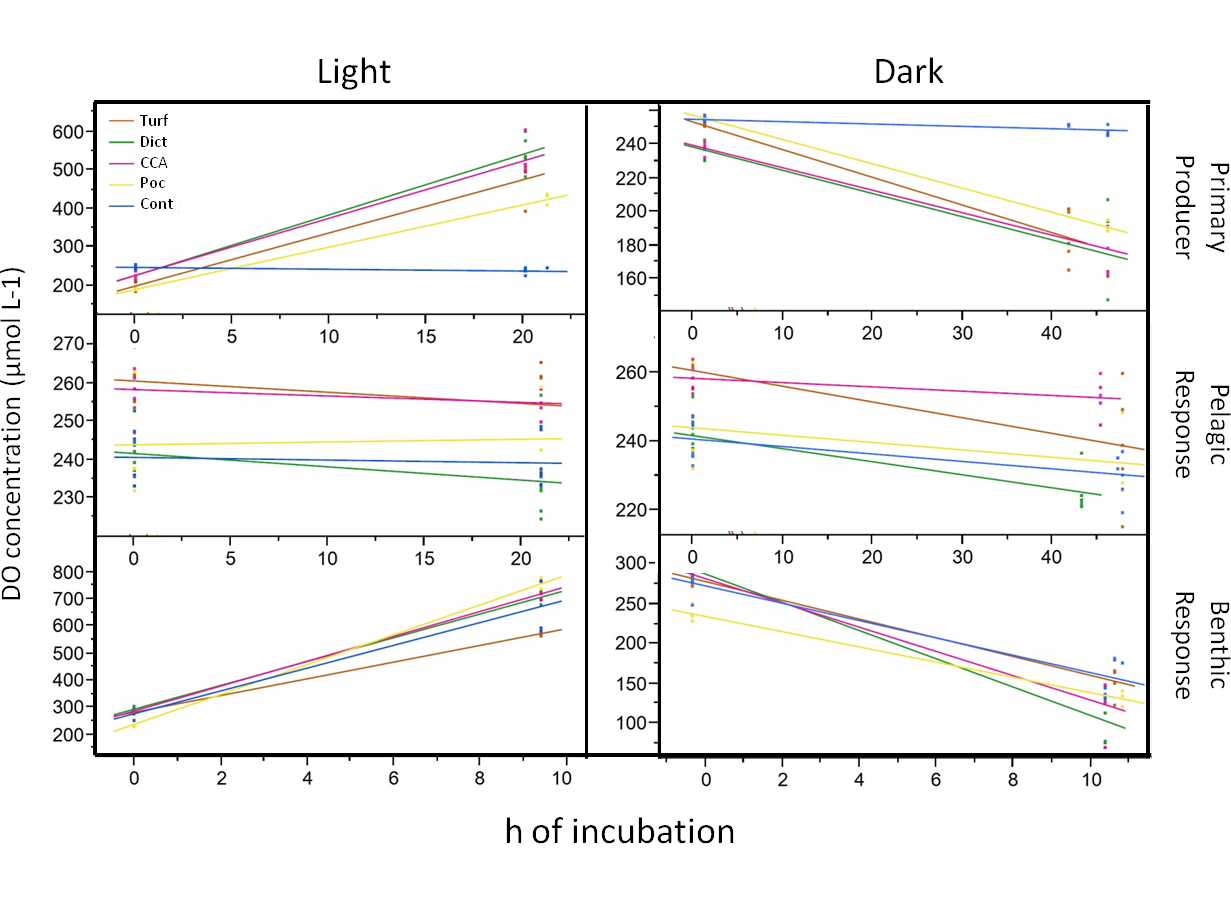

Supplement: Figure S2 — Raw values of DO measurements in dark and daylight incubations over the period exposed to the respective light conditions [file peerj-01-108-s002.png]

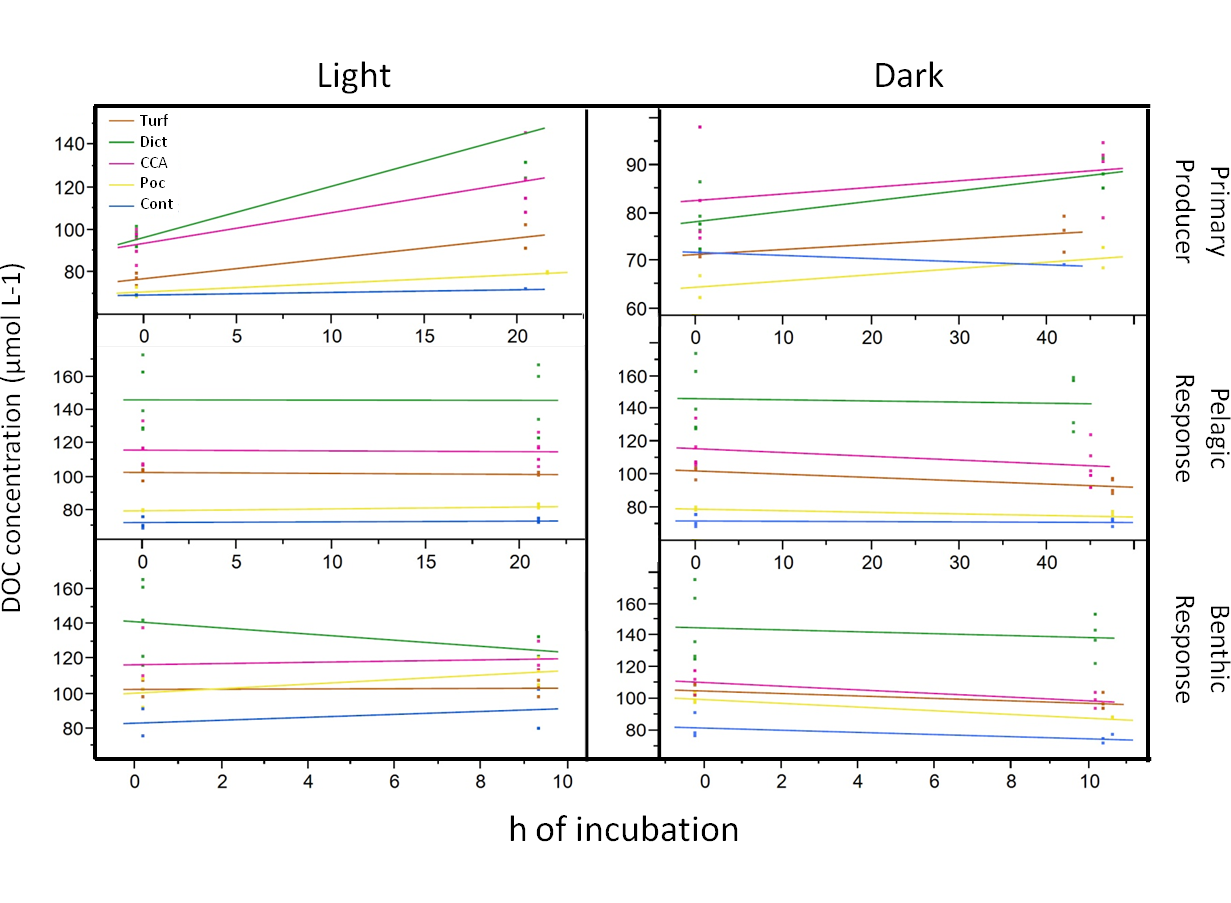

Supplement: Figure S3 — Raw values of DOC measurements in dark and daylight incubations over the period exposed to the respective light conditions. [file peerj-01-108-s003.png]
